# Novel Probable Glance at Inflammatory Scenario Development in Autistic Pathology

**DOI:** 10.3389/fpsyt.2021.788779

**Published:** 2021-12-22

**Authors:** Aida A. Harutyunyan, Hayk A. Harutyunyan, Konstantin B. Yenkoyan

**Affiliations:** ^1^Department of Biochemistry, Yerevan State Medical University After Mkhitar Heratsi, Yerevan, Armenia; ^2^Laboratory of Neuroscience, Cobrain Center, Yerevan State Medical University After Mkhitar Heratsi, Yerevan, Armenia

**Keywords:** autism spectrum disorders, lead, VDR Taq, MAO-A, energy metabolism, mitochondria, monocytes, TNF-alfa

## Abstract

Autism Spectrum Disorder (ASD) is characterized by persistent deficits in social communication and restricted-repetitive patterns of behavior, interests, or activities. ASD is generally associated with chronic inflammatory states, which are linked to immune system dysfunction and/or hyperactivation. The latter might be considered as one of the factors damaging neuronal cells. Several cell types trigger and sustain such neuroinflammation. In this study, we traced different markers of immune system activation on both cellular (immune cell phenotypes) and mediatory levels (production of cytokines) alongside adverse hematology and biochemistry screening in a group of autistic children. In addition, we analyzed the main metabolic pathways potentially involved in ASD development: energy (citric acid cycle components), porphyrin, and neurotransmitter metabolism. Several ASD etiological factors, like heavy metal intoxication, and risk factors—genetic polymorphisms of the relevant neurotransmitters and vitamin D receptors—were also analyzed. Finally, broad linear regression analysis allowed us to elucidate the possible scenario that led to the development of chronic inflammation in ASD patients. Obtained data showed elevated levels of urinary cis-aconitate, isocitrate, alfa-ketoglutarate, and HMG. There were no changes in levels of metabolites of monoamine neurotransmitters, however, the liver-specific tryptophan kinurenine pathway metabolites showed increased levels of quinolinate (QUIN) and picolinate, whereas the level of kynurenate remained unchanged. Abovementioned data demonstrate the infringement in energy metabolism. We found elevated levels of lead in red blood cells, as well as altered porphyrin metabolism, which support the etiological role of heavy metal intoxication in ASD. Lead intoxication, the effect of which is intensified by a mutation of the VDR-Taq and MAO-A, leads to quinolinic acid increase, resulting in energy metabolism depletion and mitochondrial dysfunction. Moreover, our data backing the CD4+CD3+ T-cell dependence of mitochondrial dysfunction development in ASD patients reported in our previous study leads us to the conclusion that redox-immune cross-talk is considered a main functional cell damaging factor in ASD patients.

## Highlights

- Lead poisoning is a potential etiological factor in ASD development.
- Lead intoxication and mutations of the VDR Taq and MAO-A all lead to increases in quinolinic acid level.
- Disordered porphyrin metabolism supports the etiological role of heavy metal intoxication
- Kynurenine pathway and citric acid cycle obstacles ultimately lead to reduction of energy metabolism.
- Mitochondrial dysfunction and reactive oxygen species (ROS) overproduction eventually trigger T-cell dependent inflammation.
- Activated T-helpers via TNF-α lead to persistent chronic inflammation.

## Introduction

Autism and its related neurodevelopmental disorder (Autism Spectrum Disorder-ASD) are clinically heterogeneous pathologies, which are caused by a number of factors. Such heterogeneity makes it difficult to single out individual causal elements of this disease(s). However, certain genetic and environmental triggers are already suggested, including molecular/genetic changes affecting brain development (1). According to CDC studies, the number of children diagnosed with ASD has increased over the last decade and ASD currently affects as many as 1 out of 54 individuals (2).

Clinical signs of ASD are frequently present at 3 years of age and recent prospective studies in toddlers indicate that abnormalities in social, communicative, and play behavior, which may represent early indicators of autism, can be detected as early as at 14 months of age (3). Abnormalities in language development, delayed mental development, and epilepsy are frequent problems in the clinical profiles of patients with autism, and some patients may exhibit features of clinical regression, in which neurodevelopmental milestones are lost and/or other clinical signs are worsened (4). Cases of ASD are clinically heterogeneous and can be associated in up to 10% of patients with well-described neurological and genetic disorders, such as tuberous sclerosis, fragile X, Rett's and Down syndromes, although in most patients the causes are still unknown (5, 6).

Continuing investigations for a neurobiological basis of ASD support the view that genetic, environmental/toxic (heavy metal intoxication, particularly by lead and mercury), neurological, metabolic, digestive, and immunological factors contribute to its etiology. In particular, there is evidence that suggests an association between ASD and neuroinflammation in anterior regions of the neocortex (7, 8), and areas related to cognitive function appear to be affected by inflammation due to activation of microglia and astrocytes (9). *In vivo* measurements of structural brain changes with magnetic resonance imaging in ASD patients detected gray matter loss in the orbitofrontal cortex and impairment of cognitive functions mediated by the orbitofrontal–amygdala circuit (10, 11). Furthermore, markers of oxidative stress are elevated in the orbitofrontal cortex in post-mortem samples of ASD patients and in blood of autistic children (12, 13).

A strong inflammatory state associated with ASD is currently increasingly reported in literature (14). The inflammatory states observed in ASD patients are mostly related to immune system dysfunction (15). Increased cytokine levels (IL-1β, IL-6, IL-8, and IL-12p40), among others, were found to be associated with impairments in stereotypical behaviors, suggesting that dysfunctional immune responses could affect core behaviors in ASD (16). Over-production of pro-inflammatory cytokines was also demonstrated *in vitro*, in cultured and stimulated peripheral blood monocytes from ASD children (17). Both Th1 and Th2 cytokines have been reported to be increased in ASD children (18).

As reported by Siniscalco and coauthors, plasma cytokine profiles were different between the two grades of severity of ASD (moderate and mild, according to the Childhood Autism Rating Scale (CARS) test) (19). IL-12p40 levels were higher in the patients with mild disease severity whereas tumor necrosis factor alpha (TNF-α) appeared to be more pronounced in the patients with moderate severity (20, 21). It has been suggested that TNF-α levels positively correlate with ASD severity (as tested by Autism Behavior Checklist, ABC), thus being an indicator of ASD phenotype (19).

The immune system is closely related to the pro-/antioxidant homeostasis. The importance of ROS in immune defense is exemplified by their generation and release in the form of an “oxidative burst” by phagocytic cells (e.g., neutrophils and macrophages). Recent studies revealed numerous immunologic abnormalities among children with autism including elevated generation of free radicals in lymphocytes (22). In our previous study, we demonstrated that persistent inflammation shown in ASD patients may lead to depletion of the respiratory burst capability in neutrophils, and accumulation of damage and pathological change resulting in disability and disease (13).

Earlier, it was shown that ASD pathology is also accompanied by mitochondrial dysfunction. As ROS can cause mitochondrial dysfunction, oxidative stress may be a key mechanism by which mitochondria are damaged by factors linked to ASD such as pro-oxidant environmental toxicants (23–26). Furthermore, dysfunctional mitochondria can create a self-perpetuating cycle of progressive damage that amplifies functional deficits. Indeed, mitochondria are a major source of ROS as well as the target for ROS-mediated damage (27). So, environmental pro-oxidants may alter the electron transport chain complex I, thus producing higher levels of ROS (28). In turn, ROS can inhibit electron transport chain function and MnSOD activity causing further mitochondrial dysfunction (29). However, a link between bioenergetics and the immune response in autism has not been explored yet.

While our previous study was focused on the role of imbalanced red-ox states in the pathogenesis of autism (13), the current report is dedicated to the immunological aspects of ASD pathology development. Analysis of immune system activation markers (immune cell phenotype and cytokines) is complemented with adverse hematology and biochemistry screening. In addition, we traced energy producing (citric acid cycle) components, neurotransmitter and porphyrin content, genetic polymorphisms of relevant neurotransmitters, and vitamin D. Heavy metal content as a main ethological factor in ASD has been assessed. Finally, broad linear regression analysis allowed us to elucidate the possible scenario that led to the development of chronic inflammation in ASD patients.

## Methods

### Participants

Twenty-four preschoolers aged 3–6 years old participated in the study. Among them 12 children (age range: 3–6 years old) diagnosed with ASD/MD who had not previously received any type of treatment were recruited for this study (autistic group). All children met the DSM-IV criteria for Autistic Disorder and this diagnosis was also corroborated by psychologists using the Autism Diagnostic Interview–Revised (ADI-R) and the Autism Diagnostic Observation Schedule (ADOS). Children with PDD-NOS, Asperger syndrome, seizure disorder, current ear infection, uncontrolled asthma, inability to equalize ear pressure, fragile X syndrome, and ongoing treatment with chelation medication were excluded from participation in this study. Written informed consent was obtained from the parents and, when possible, the child. Unaffected 12 siblings of the same age (age range: 3–6 years old) with no history of behavioral or neurologic abnormalities according to parents' reports, were recruited for the comparison group (control group). The protocol was approved by the Ethics Committee of YSMU, and all parents signed informed consent.

### Sample Collection and Preparation

Blood samples from fasting patients (10.0 mL) were collected before 9:00 a.m. into two (5.0 ml per tube) EDTA, trace element free, tubes (royal blue top; BD Vacutainer, Franklin Lakes, NJ). Aliquot of the whole blood was used for complete blood count assay. Tubes were centrifuged for 1,500 × g for 15 min at 4°C within 30 min of initial blood draw. After centrifugation, plasma, including the white buffy layer, was separated using disposable pipettes and centrifuged again under the same conditions. Plasma samples were transferred into the new Eppendorf tubes for further biochemical tests. Cell pellet was resuspended in PBS (10 mM; pH 7.4) and immediately used for respiratory burst analysis and single nucleotide polymorphism (SNP) assay. RBC pellet formed after the first centrifugation was used for trace element measurements (arsenic, cadmium, lead, mercury, and thallium). To test energy production (citrate, cis-aconitate, isocitrate, alfa-ketoglutarate, succinate, fumarate, malate, and hydroxyethylglutarate), neurotransmitter metabolism (vanilmandelate, homovanillate, 5-hydroxyindoleacetate, kynurenate, QUIN, and picolinate) biomarkers, and porphyrin content of urine, the first morning urine samples (10.0 mL) were collected into sterile plastic containers. Urine common biochemistry was also tested. Barcoded plasma, red blood cells, and urine aliquots were frozen at −70°C until measurements were taken.

### CBC Count

CBC was analyzed in EDTA-treated whole blood samples using the Sysmex XS 500i fully automated hematology analyzer (Sysmex Corporation, Kobe, Japan).

### Blood Plasma Biochemistry

Biochemical parameters: total protein (TP), C-reactive protein (CRP), vitamin B12, vitamin D, aminotransferases (ALAT and ASAT), alkaline phosphatase (ALP), gamma-glutamyl transferase (GGT), glucose, bilirubin (BIL-T and BIL-D), creatinine, and urea were measured in the automatic biochemical analyzers Roche Cobas C311 and Cobas E411 (Roche Cobas, Swiss) using appropriate test kits purchased from the manufacturer.

### Urine Measurements

Routine biochemical urine tests were performed using Cobas Urisys 1100 analyser (Roche Cobas, Swiss) using appropriate test strips. Energy production (citric acid cycle intermediates—citrate, cis-aconitate, isocitrate, alfa-ketoglutarate, succinate, fumarate, malate, and hydroxymethylglutarate), neurotransmitter metabolism (quinolinate, picolinate, vanilmandelate, homovanillate, 5-hydroxyindolacetate, and kynurenate) biomarkers, and porphyrin content (uroporphyrins, heptacarboxylporphyrins, hexacarboxylporphyrins, pentacarboxylporphyrins, coproporphyrin I concentration, coproporphyrin III, total porphyrins, precoproporphyrin I, precoproporphyrin II, precoproporphyrin III concentration, and total precoproporphyrins) in the urine were performed using LC/MS-MS spectrophotometry (Organix Comprehensive Profile; Metametrix, Inc, Duluth, GA). Urine samples were collected in accordance with instructions provided by Metametrix Clinical Laboratory. In brief, participants were instructed to void (empty bladder) before going to bed and then place a collection basin over their home toilets. Participants used a pipette to place 12 mL of their first morning sample plus any overnight sample into a test tube. At the laboratory, urine samples were frozen and then shipped to Metametrix Clinical Laboratory for biochemical analyses.

### Packed Red Blood Cell Elements Assay

Potentially toxic mineral elements (arsenic, cadmium, lead, mercury, and thallium) were measured by Doctor's Data (St. Charles, IL, USA) in packed RBC. Packed red blood cells were spun for 15 min in a centrifuge at 1,500 × g, the plasma and buffy coat were removed, and the remaining packed red blood cells were submitted for testing. Elemental analysis was performed after digesting an aliquot of sample using a temperature-controlled microwave digestion system (Mars5; CEM Corp; Matthews, SC). The digested sample was analyzed by Inductively Coupled Plasma-Mass Spectrometry (ICP-MS) (Elan DRCII; Perkin Elmer Corp; Shelton, CT). Results were verified for precision and accuracy using controls from Doctor's Data.

### Flow Cytometry Assay for Lymphocyte Phenotyping

Peripheral blood mononuclear cells (PBMC) were separated from heparinized whole blood by Ficoll-Hypaque (histopaque) (Sigma Chemical Co., St. Louis, MO, USA) density gradient centrifugation. Expression of the CD3, CD4, CD8, CD14, CD19, and CD56 cells was assessed by incubating one million PBMC in 100 ml total volume with appropriate antibodies conjugated to peridinin chlorophyll-a protein (PerCP-0.12 mg) (BD/Pharmingen, San Diego, CA, USA), or 20 ml phycoerythrin (eBioscience, San Diego, CA, USA) for 40 min. Cells were then washed and incubated with 0.5 mg fluorescein isothiocyanate-conjugated anti-mouse IgM (eBioscience) for an additional 30 min, fixed with 1% paraformaldehyde, and analyzed on a Becton Dickinson FACSCalibur flow cytometer with CellQuest and Attractors software (Becton Dickinson, San Jose, CA, USA). Isotype controls were PerCP-conjugated mouse IgG1k (BD Pharmingen), phycoerythrin-conjugated mouse IgG1k, and mouse IgMk control (eBioscience).

### Flow Cytometry Assay for TNF-α and IL-10

PBMC were surface-stained by 0.3 μg fluorescein isothiocyanate (FITC)-conjugated anti-TNF-α (eBioscience, San Diego, CA, USA) or IgM-FITC isotype control and fixed in 1% paraformaldehyde. Stained cells were incubated with 1% FACS permeabilizing solution (Becton Dickinson) and incubated with phycoerythrin (PE)-conjugated anti-human IL-10 (eBioscience, San Diego, CA, USA) or matched isotype control. Cells were fixed in 1% paraformaldehyde and subjected to FACS analysis as described above.

### Flow Cytometry Assay of Respiratory Burst

Spontaneous and N-formyl-L-methionyl-L-leucyl-L-phenylalanine (fMLP)-induced respiratory burst of monocytes was determined by flow cytometry using Cayman's Neutrophil/Monocyte Respiratory Burst Assay Kit according to manufacturer instructions (Cayman Chemical, Ann Arbor, MI, USA). The flow cytometry analyses of respiratory burst were based on dihydrorhodamine 123 (DHR) oxidation to the fluorescent rhodamine 123 (RHO). Respiratory burst was activated in monocytes by fMLP (100 nM, 10 min, 37°C) (Sigma, St. Louis, USA). Samples without any external stimulus were used to measure spontaneous respiratory burst. Gated neutrophil population was monitored by determining the RHO relative fluorescence intensities (expressed as a mean channel number—MCN) on FACSCaliburTM instrument using CellQuestTM software (BD Biosciences, Heidelberg, Germany).

### Neurotransmitter Related SNP Genotyping

Three genes (five polymorphisms), encoding for essential proteins involved in neurotransmitter metabolism, were studied: catechol-O-methyltransferase (COMT V158/M and COMT H62H), vitamin D receptor (VDR Taq and VDR Fok), and monoamine oxidase A (MAO-A R297R). The genomic location, known nonsynonymous coding variants, and the number of single nucleotide polymorphism (SNP) markers were genotyped at each locus. SNPs were selected to efficiently assay common variation in the genes of interest. SNPs were genotyped on the Illumina BeadArray platform using the Golden Gate genotyping technology as part of a 384-SNP customized assay. SNPs were genotyped by Holistic Health International, LLC (279 Walkers Mills Road, Bethel, ME). Genome data were uploaded in dbSNP repository, Variation File submission: SUB10619200.

### Infectious Diseases Assay

Antibodies (IgM/IgG) to Chlamydia trachomatis, Toxoplasma gondii, Herpes simplex virus (HSV-1,2), and Cytomegalovirus (CMV) were tested by chemiluminescence assay using Cobas E411 (Roche Cobas, Swiss), using appropriate test kits purchased from the manufacturer. Additionally, CMV and Epstein-Barr virus (EBV) were screened by the conventional PCR method using GeneAmp 9700 thermal cycler (Applied Biosystems, USA). DNA samples were obtained from peripheral blood, monocytes, and throat swabs. PCR detection kits for the aforementioned infections were purchased from DNA-Technology (DNA-Technology LLC, Russia).

### Data Analysis

Data analysis was carried out in GrafPad InStat software Version 3.10 (GraphPad Software, San Diego, CA). Non-parametric Wilcoxon rank sum test was applied. ASD patient data were compared to the appropriate control (comparison) group or reference values (hypothetical median). Reference values were obtained from the international database (Reference Ranges for Adults and Children. Pre-Analytical Considerations. Roche Diagnostics GmbH, Mannheim, 2008; Organix Comprehensive Profile; Metametrix, Inc, Duluth, GA; Doctor's Data, St. Charles, IL, USA; SNPedia). Categorical variables (SNP data, PCR test results) were summarized in Odds Ratio (OR). Pearson correlation coefficients were computed to determine inter-metabolite correlations. *P* < 0.05 were used to indicate statistical significance.

## Results

### Complete Blood Count, Clinical Biochemistry, and Complete Urine Test in ASD Patients

**Figure 1:**
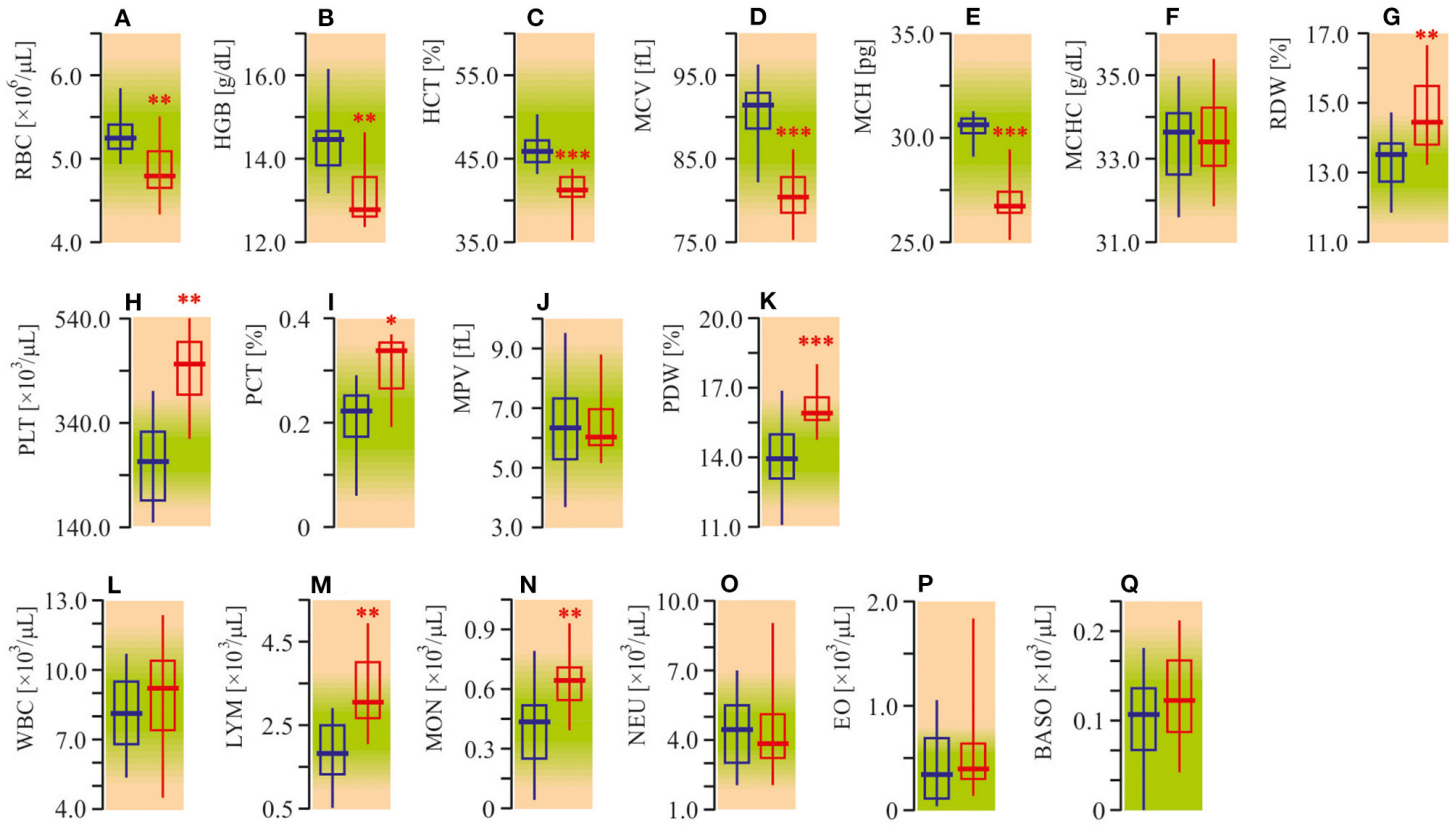
Complete blood count (CBC) of ASD patients. **(A)** Red blood cell (RBC) count. **(B)** Hemoglobin concentration (HGB). **(C)** Hematocrit. **(D)** Mean corpuscular volume (MCV). **(E)** Mean corpuscular hemoglobin (MCH). **(F)** Mean corpuscular hemoglobin concentration (MCHC). **(G)** Red blood cell distribution width (RDW). **(H)** Platelets count (PLT). **(I)** Plateletcrit (PCT). **(J)** Mean platelet volume (MPV). **(K)** Platelet distribution width (PDW). **(L)** White blood cell count (WBC). **(M)** Lymphocyte count (LYM). **(N)** Monocyte count (MON). **(O)** Neutrophil count (NEU). **(P)** Eosinophil count (EO). **(Q)** Basophil count (BASO). CBC was analyzed in EDTA treated whole blood samples using the Sysmex XS 500i fully automated hematology analyzer (Sysmex Corporation, Kobe, Japan). Blue graph—control data, red graph—ASD data. Non-parametric Wilcoxon rank sum test was applied. ASD patients' data were compared to the appropriate control group. *P* < 0.05 were used to indicate statistical significance. *P < 0.05, **P < 0.01, ***P < 0.001.

Diminished red blood cell (RBC) parameters were observed in autistic patients (Figures 1A–E). The medians of RBC count, hemoglobin (HGB) content, hematocrit (HCT), mean corpuscular volume (MCV), and mean corpuscular hemoglobin (MCH) were 10–15% lower in ASD patients compared to the healthy controls (*P* < 0.01). Mean corpuscular hemoglobin concentration (MCHC) was unchanged (*P* < 0.05, Figure 1F). Meanwhile, distribution width (RDW) was about 8% (*P* < 0.01) higher (Figure 1G). At the same time, the peripheral blood of autistic children was characterized by a 50% enhanced (*P* < 0.01) platelet (PLT) count (Figure 1H), increased plateletcrit (PCT) (*P* < 0.05) and platelet distribution width (PDW) (*P* < 0.001, Figures 1F,K), unchanged mean platelet volume (MPV) (*P* > 0.05, Figure 1J). The medians of lymphocytes (LYM) and monocytes (MON) in ASD patients were also higher by 64% (*P* < 0.001) and 20% (*P* < 0.01) respectively as compared to the appropriate control (Figures 1M,N). Simultaneously, the number of total white blood cells (WBC), as well as neutrophils (NEU), eosinophils (EO) and basophils (BASO) were not significantly altered (*P* > 0.05, Figures 1L,O–Q).

**Figure 2:**
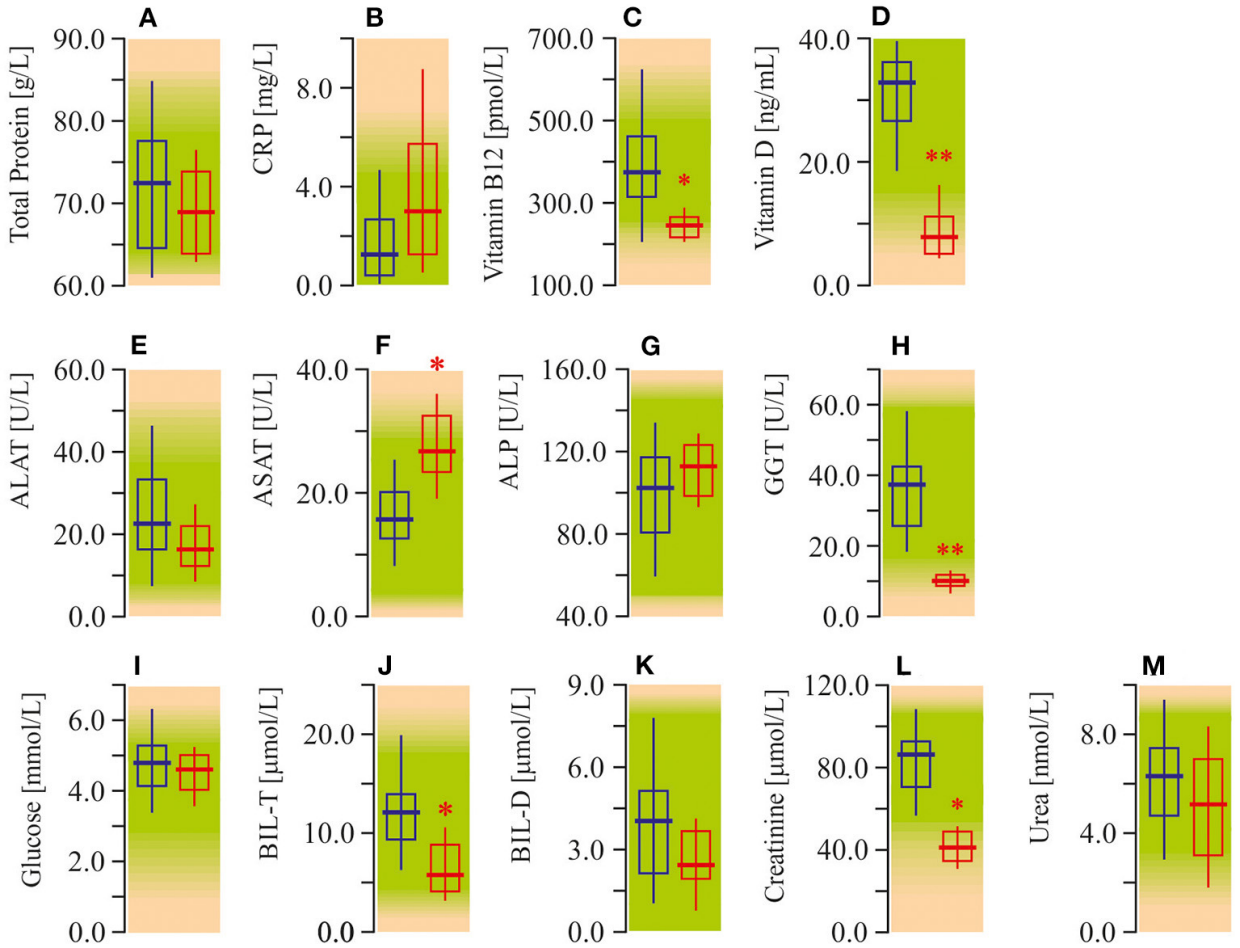
Some biochemical tests in peripheral blood plasma of ASD patients. **(A)** Total protein concentration (TP). **(B)** C-reactive protein content (CRP). **(C)** Vitamin B12 content. **(D)** Vitamin D content. **(E)** Alanine aminotransferase activity (ALAT). **(F)** Aspartate aminotransferase activity (ASAT). **(G)** Alkaline phosphatase activity (ALP). **(H)** Gamma glutamyl transferase activity (GGT). **(I)** Glucose concentration. **(J)** Total bilirubin concentration (BIL-T). **(K)** Direct bilirubin concentration (BIL-D). **(L)** Creatinine concentration. **(M)** Urea concentration. Biochemical parameters were measured in an automatic biochemical analyzers Roche Cobas C311 and Cobas E411 (Roche Cobas, Swiss) using appropriate test kits purchased from manufacturer. Blue graph—control data, red graph—ASD data. Non-parametric Wilcoxon rank sum test was applied. ASD patients' data were compared to the appropriate control group. *P* < 0.05 were used to indicate statistical significance. ^*^P < 0.05, ^**^P < 0.01.

The study of serum biochemistry parameters demonstrates diminished content of vitamin B12 (−50%; *P* < 0.05) and especially vitamin D (−75%; *P* < 0.01; Figures 2C,D). In addition, we also recorded enhanced (40%; *P* < 0.05) activity of ASAT. However, GGT indeed showed a significant decrease, as did BIL, and creatinine values. At the same time, liver tests, such as ALAT, and ALP were not significantly different from the control group (Figures 2E,G,H,J,K). In other tests of serum biochemistry, as well as urine biochemistry, parameters remained within reference limits (Figure 2; Table 1).

**Table 1:** Urine biochemistry.

**Parameter**	**Reference range**	**ASD patients**		
		**Mediana**	**95% CI**	***P*-value**
Specific Gravity [g/mL]	1.005–1.035	1.025	1.019–1.026	> 0.05
pH	4.50–8.00	6.00	5.99–6.76	> 0.05
Glucose [mg/dL]	0	0		
Bilirubin [mg/dL]	0	0		
Urobilinogen [mg/dL]	<0.1	0		
Keton [mg/dL]	0	0		
Protein [mg/dL]	0	0		
Nitrite [mg/dL]	0	0		
RBC [10^3^/uL]	0	0		
WBC [Cell/uL]	0	0		

### Potential Etiological and Risk Factors of the Autistic Pathology

Among the many known etiological factors leading to autistic pathology development, we decided to investigate the levels of potentially toxic elements in packed RBC, SNP of enzymes responsible for the metabolism of some neurotransmitters, as well as incidents of some potential infectious diseases.

### Heavy Metal Changes

**Figure 3:**
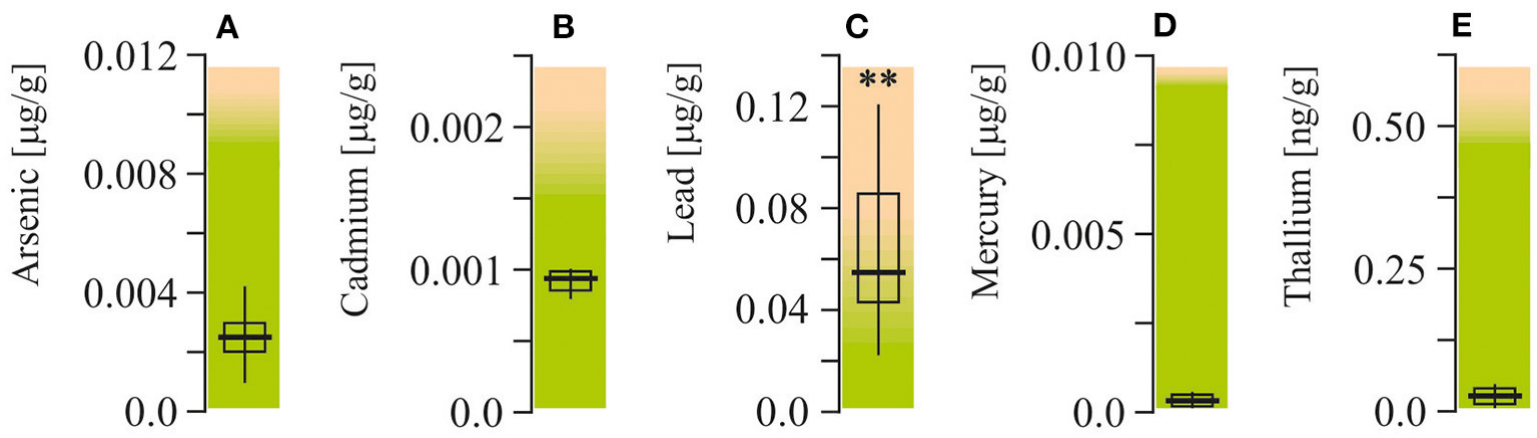
Potentially toxic mineral elements content in packed red blood cell of ASD patients. **(A)** Arsenic concentration. **(B)** Cadmium concentration. **(C)** Lead concentration. **(D)** Mercury concentration. **(E)** Thallium concentration. Potentially toxic mineral elements were measured by Doctor's Data (St. Charles, IL, USA). Green area-reference range. Non-parametric Wilcoxon rank sum test was applied. ASD patients' data were compared to the appropriate reference values—hypothetical median. *P* < 0.05 were used to indicate statistical significance. ^**^P < 0.01.

Arsenic, cadmium, lead, mercury, and thallium were measured in packed red blood cells (Figure 3). Among all of these elements, lead was the only one with levels exceeding the upper reference limit (more than twofold, *P* < 0.001).

**Figure 4:**
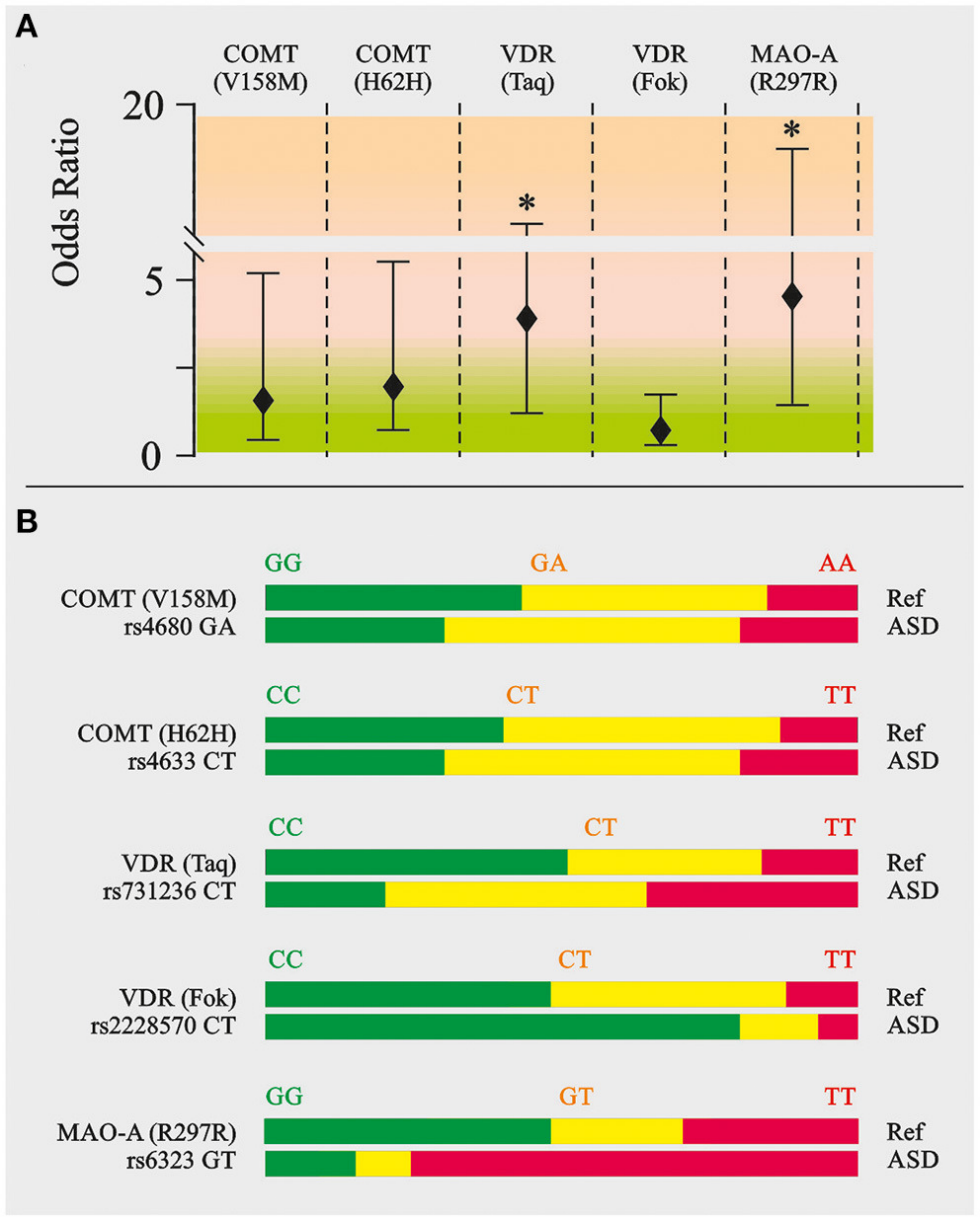
Neurotransmitter related SNP genotyping in ASD patients. Catechol-O-methyltransferase (COMT V158/M and COMT H62H), vitamin D receptor (VDR Taq and VDR Fok), monoamine oxidase A (MAO-A R297R). **(A)** Odds ratio calculation. **(B)** Appropriate allele ratio. The genomic location, known nonsynonymous coding variants and the number of single nucleotide polymorphism (SNP) markers genotyped at each locus. SNPs were selected to efficiently assay common variation in the genes of interest. SNPs were genotyped on the Illumina Bead Array platform using the Golden Gate genotyping technology as part of a 384-SNP customized assay. SNPs was genotyped by the Holistic Health International (279 Walkers Mills Road, Bethel, ME). Green, homozygous wild type; Yellow, heterozygous; Red, homozygous mutated. ^**^P < 0.01.

*SNPs*. Mutated allele ratios of catechol-O-transferase (COMT V158M-GA and H62H-*CT*) and vitamin D receptor (VDR Fok-*CT*) in autistic children and the healthy group were found to be very similar (Figure 4). However, the ratios of *T*-mutated allele of VDR Taq-*CT* and monoamine oxidase A (MAO-A R297R-*GT*), *OR* = 3.71 and 4.89, respectively (*P* < 0.05), were found to be higher in ASD patients.

### Infectious Diseases

Several congenital pathogens were screened in ASD patients (Chlamydia trachomatis, Toxoplasma gondii, HSV-1,2, EBV, and CMV). Neither the immunochemiluminescent test nor PCR detected any infections (excluding single cases) except CMV as shown by the detected antibodies (IgG) to CMV (208.6 AU/mL; *P* < 0.01) with high avidity (97.9%; *P* < 0.01) (Table 2). PCR analyses for CMV and EBV were done in all possible targets—blood, monocytes as well as throat swabs.

**Table 2:** Infection disease assay.

**Parameter**	**Method**	**Reference range**	**ASD patients**			
			**Odds ratio**	**Mediana**	**95% CI**	***P*-value**
Chlamydia trakhomatis	IgG (AU/mL)	<9		1.61	1.17–2.84	> 0.05
Toxoplasma gondii	IgG (AU/mL)	<2		0.2	0.08–0.45	> 0.05
HSV-1,2	IgM (AU/mL)	<8		4.77	3.57–7.46	> 0.05
	IgG (AU/mL)	<8		1.10	0.21–5.46	> 0.05
CMV	PCR (Blood)		1.0			> 0.05
	PCR (Monocytes)		1.0			> 0.05
	PCR (Throat swab)		2.3			> 0.05
	IgM (AU/mL)	<6		0.18	0.14–0.32	> 0.05
	IgG (AU/mL)	<6		208.6	103.9–765.3	<0,01
	IgG Avidity (%)	<50		97.9	96.0–98.9	<0,01
EBV	PCR (Blood)		1.0			> 0.05
	PCR (Monocytes)		3.8			> 0.05
	PCR (Throat swab)		2.7			> 0.05

### Energy Production, Neurotransmitter Metabolism Biomarkers, and Porphyrin Content in the Urine

#### Citric Acid (Krebs) Cycle Intermediates

**Figure 5:**
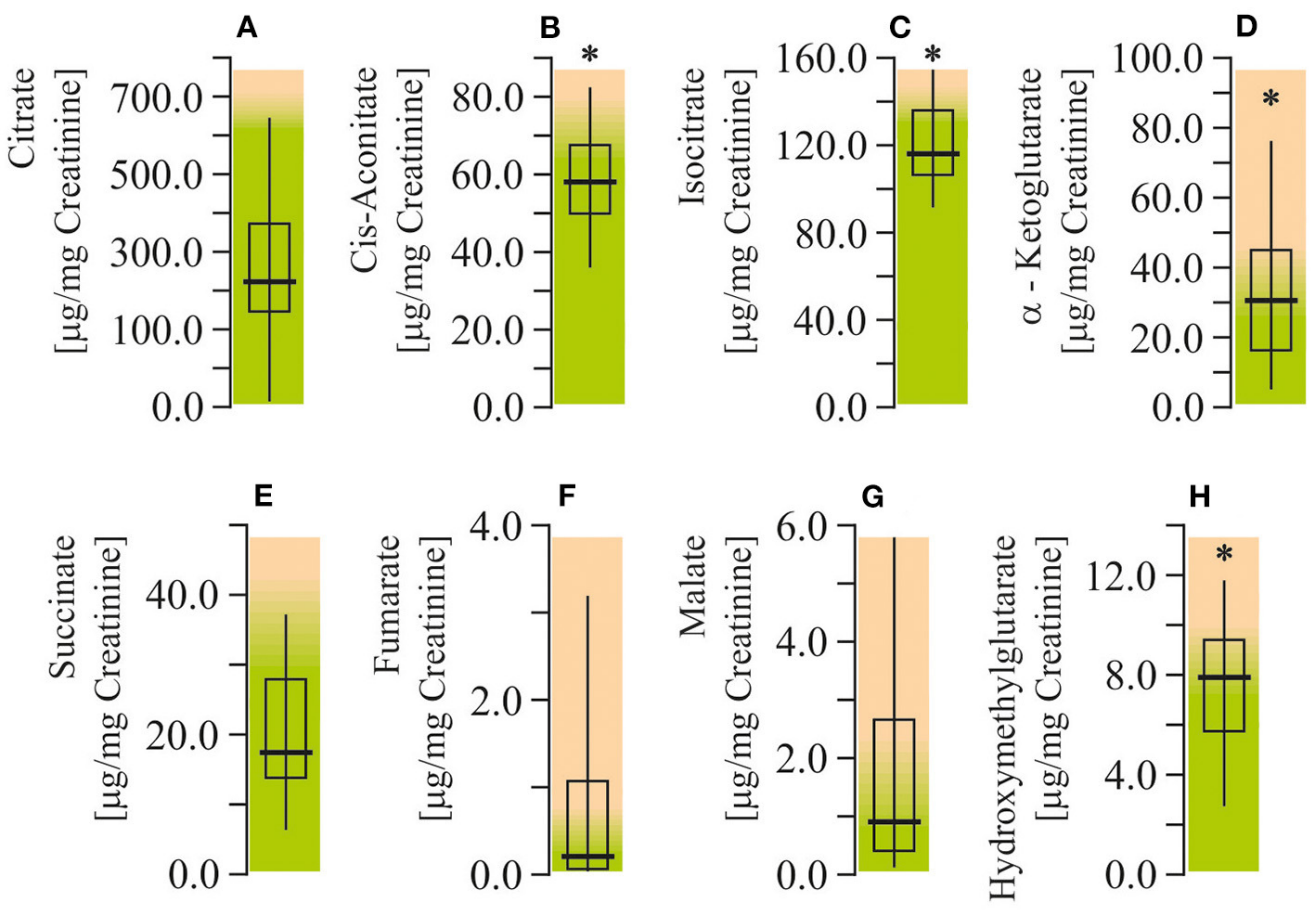
Energy production biomarkers assay in urine of ASD patients. **(A)** Citrate concentration. **(B)** Cis-aconitate concentration. **(C)** Isocitrate concentration. **(D)** α-Ketglutarate concentration. **(E)** Succinate concentration. **(F)** Fumarate concentration. **(G)** Malate concentration. **(H)** Hydroxymethylglutarate concentration. Energy production biomarkers content in the urine were performed using LC/MS-MS spectrophotometry (Organix Comprehensive Profile; Metametrix, Inc., Duluth, GA). Green area-reference range. Non-parametric Wilcoxon rank sum test was applied. ASD patients' data were compared to the appropriate reference values—hypothetical median. *P* < 0.05 were used to indicate statistical significance. ^*^P < 0.05.

The levels of citrate, cis-aconitate, isocitrate, alfa-ketoglutarate, succinate, fumarate, malate, and hydroxymethylglutarate (HMG) were measured to assess citric acid cycle function in ASD patients. The urinary levels of cis-aconitate, isocitrate, alfa-ketoglutarate, and HMG were found to be elevated (by 55–76%; *P* < 0.05) in autistic children (Figure 5). All of the indicated metabolites are components of the first stage of the citric acid cycle: from citrate to succinate.

#### Neurotransmitter Metabolites

**Figure 6:**
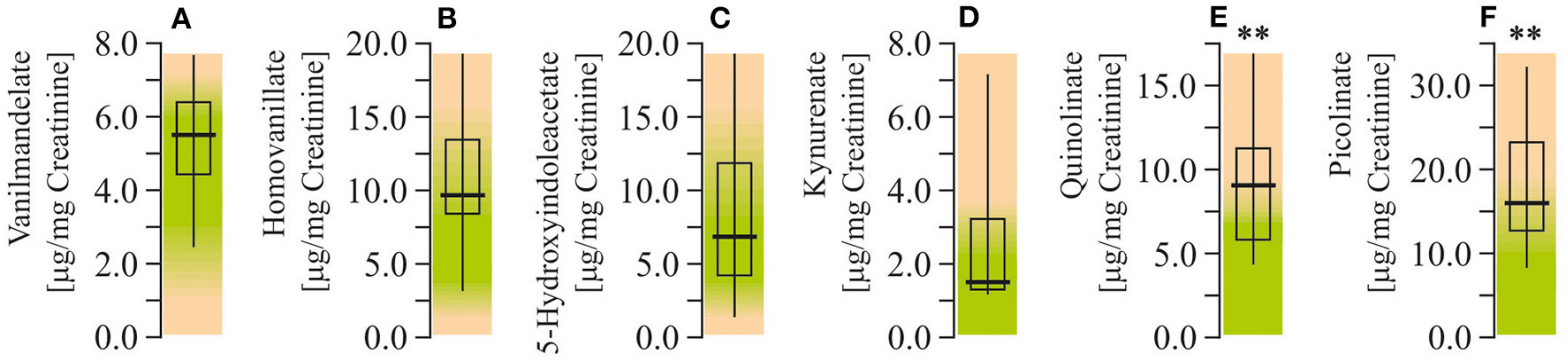
Neurotransmitter metabolism biomarkers assay in urine of ASD patients. **(A)** Vanilmandelate concentration. **(B)** Homovanillate concentration. **(C)** 5-Hydroxyindolacetate concentration. **(D)** Kynurenate concentration. **(E)** Quinolinate concentration. **(F)** Picolinate concentration. Neurotransmitter metabolism biomarkers in the urine were performed using LC/MS-MS spectrophotometry (Organix Comprehensive Profile; Metametrix, Inc., Duluth, GA). Green area-reference range. Non-parametric Wilcoxon rank sum test was applied. ASD patients' data were compared to the appropriate reference values—hypothetical median. *P* < 0.05 were used to indicate statistical significance. ^**^P < 0.01.

Study of the neurotransmitter metabolites in the urine demonstrated that there were no changes in levels of monoamine metabolites. Particularly, concentrations of vanilmandelate (epinephrine and norepinephrine degradation marker), homovanillate (dopamine degradation marker), 5-hydroxyindolacetate (serotonin marker) were within the reference ranges. At the same time, study of the liver-specific tryptophan ynurenine pathway metabolites showed increased levels of QUIN and picolinate (by 125% and 94%, respectively; *P* < 0.001), whereas the level of kynurenate remained unchanged (Figure 6).

#### Porphyrin Metabolites

**Figure 7:**
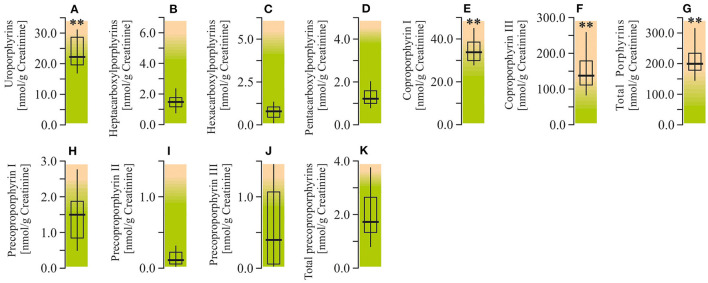
Porphyrin content in urine of ASD patients. **(A)** Uroporphyrins concentration. **(B)** Heptacarboxylporphyrins concentration. **(C)** Hexacarboxylporphyrins concentration. **(D)** Pentacarboxylporphyrins concentration. **(E)** Coproporphyrin I concentration. **(F)** Coproporphyrin III concentration. **(G)** Total porphyrins concentration. **(H)** Precoproporphyrin I concentration. **(I)** Precoproporphyrin II concentration. **(J)** Precoproporphyrin III concentration. **(K)** Total precoproporphyrins concentration. Porphyrin content in the urine were performed using LC/MS-MS spectrophotometry (Organix Comprehensive Profile; Metametrix, Inc, Duluth, GA). Green area-reference range. Non-parametric Wilcoxon rank sum test was applied. ASD patients' data were compared to the appropriate reference values—hypothetical median. *P* < 0.05 were used to indicate statistical significance. ^**^P < 0.01.

Analysis of the urine porphyrin spectra in ASD patients revealed a moderate elevation of uroporphyrins (by 47%; *P* < 0.01) and pronounced enhancement of coproporphyrin I (by 90.1%; *P* < 0.01), coproporphyrin III (by 182.7%; *P* < 0.01), and total porphyrins (by 167.4%; *P* < 0.01). At the same time, the urine levels of carboxylporphyrins and precoporphyrins in autistic children were within the normal reference values (Figure 7).

#### Immune Cell Phenotype, Phagocytosis, Cytokine Assay

**Figure 8:**
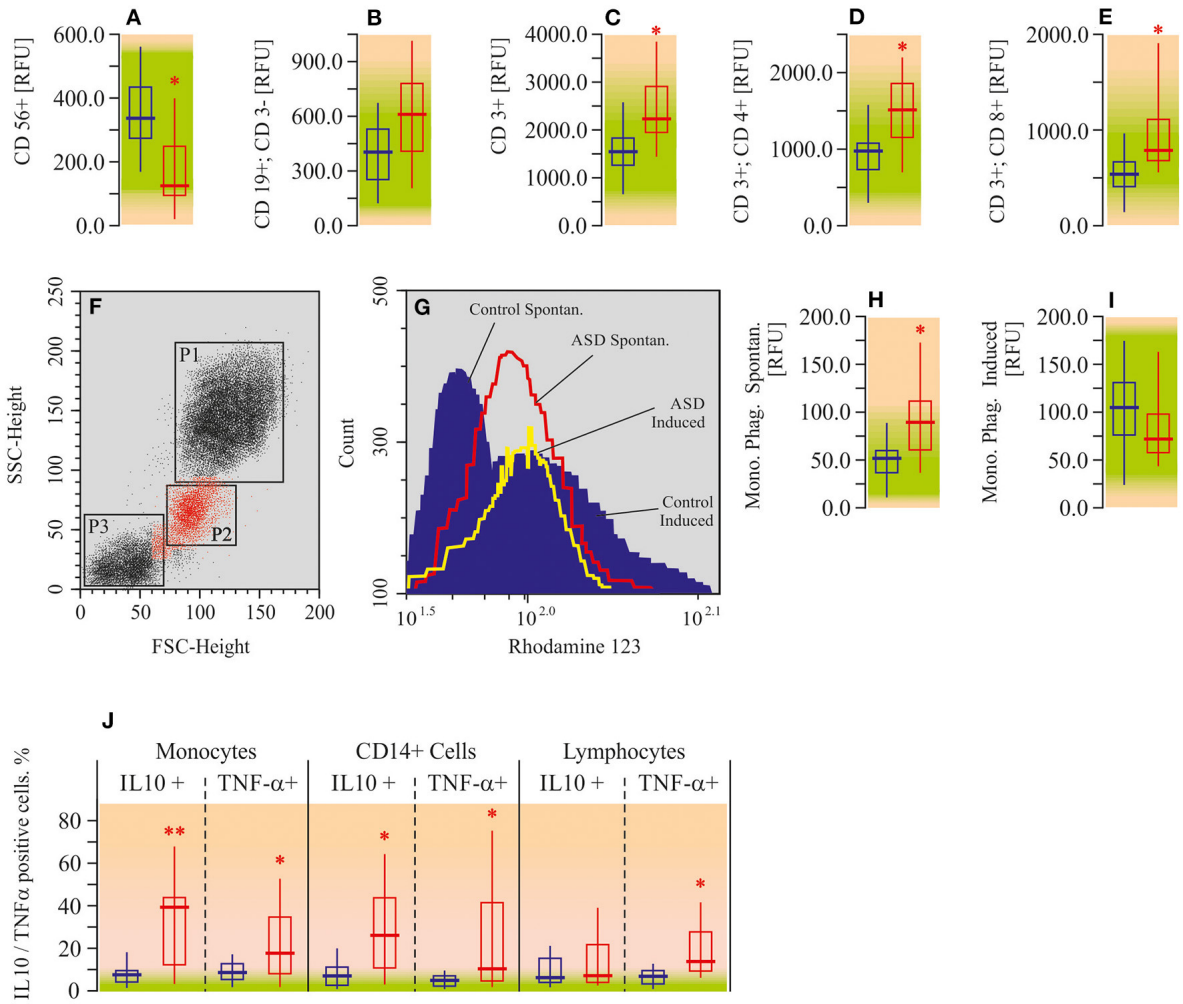
Flow cytometric analysis for immune cell phenotype, phagocytosis, and cytokine assay in peripheral blood mononuclear cells of ASD patients. **(A)** CD56 positive cell ratio (CD56+). **(B)** CD19 positive CD3 negative cell ratio (CD19+; CD3-). **(C)** CD3 positive cell ratio (CD3+). **(D)** CD3 and CD4 double positive cell ratio (CD3+; CD4+). **(E)** CD3 and CD8 double positive cell ratio (CD3+; CD8+). **(F)** Forward angle light scatter (FSC) and side scatter (SSC) segregates PMNL (gate P1), monocytes (gate P2) and lymphocytes (gate P3). **(G)** Spontaneous respiratory burst in Monocytes (red line); fMLP-induced respiratory burst in monocytes (yellow line); control data (blue area). **(H)** Monocyte phagocytosis intencity—spontaneous (Mono. Phag. Spontan.). **(I)** Monocyte phagocytosis intencity—induced by fMLP (Mono. Phag. Induced). **(J)** Flow cytometry assay for TNF-a and IL-10 in PBMC. Cells were surface-stained by 0.3 μg fluorescein isothiocyanate (FITC)-conjugated anti-TNF-α and after permeabilizing by anti-human IL-10. Flow cytometry date were analyzed on a Becton Dickinson FACSCalibur flow cytometer with CellQuest and Attractors software (Becton Dickinson, San Jose, CA, USA). Green area-reference range. Non-parametric Wilcoxon rank sum test was applied. ASD patients' data were compared to the appropriate control values. *P* < 0.05 were used to indicate statistical significance. ^*^P < 0.05, ^**^P < 0.01.

Using flow cytometry assay, we measured the number of NK-cells (CD 56+), B-lymphocytes (CD19+/CD3-), total T-lymphocytes (CD3+), helper T-cells (CD3+/CD4+), and regulatory T-cells (CD3+/CD8+) in the peripheral blood of ASD patients. All of the mentioned lymphocyte populations, except NK-cells and B-lymphocytes, were elevated in autistic patients compared to the appropriate controls (Figures 8A–E). The increase in helper T-cell levels was the most pronounced one (by 50%; *P* < 0.05), whereas the NK-cell count was found to be decreased (−50%; *P* < 0.05).

Analysis of the respiratory burst (spontaneous and fMLP-induced) of peripheral blood monocytes revealed enhancement of spontaneous phagocytosis (by 51%; *P* < 0.05) in autistic patients. Induced (by fMLP) phagocytosis was within the reference range (Figures 8F–I). We have also measured pro- (TNF-α) and anti-inflammatory (IL-10) cytokine expression in monocytes, macrophages (CD14+), and T-lymphocytes (CD3+). Both TNF-α (4–7 times; *P* < 0.05) and IL-10 (10–16 times; *P* < 0.05) were elevated in monocytes and macrophages (Figure 8J) of ASD patients, compared to the appropriate control. Lymphocytes were characterized by the increase of TNF-α only (4-fold; *P* < 0.05). Alterations of IL-10 in lymphocytes were not statistically significant.

#### Linear Regression Analysis

In order to clarify the relationships between the measured parameters and to deduce the possible way of ASD pathology development, we computed Pearson correlation coefficients. The following correlations were investigated: 1. *relations* between the potential ASD etiological and risk factors and the changes in T-helper cells; 2. *correlation* of mitochondrial damage markers with the T-helper cells; 3. *the pathway* of effector cell (monocytes respiratory burst) activation; and 4. *Correlation* between lead poisoning and precoproporphyrin appearance in urine of ASD patients.

**Figure 9:**
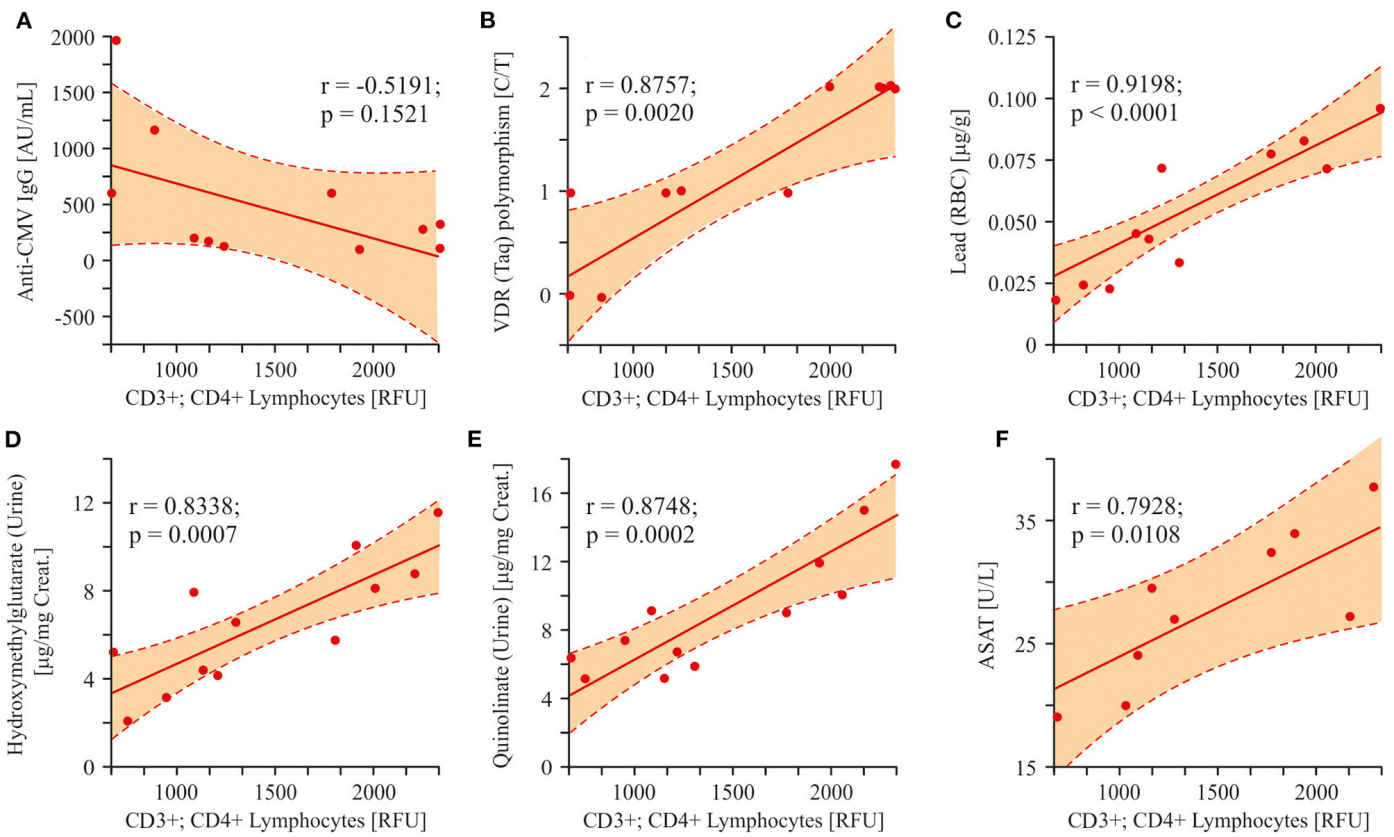
Linear regression analysis (Pearson product-moment correlation) of the interrelation between CD3+CD4+ lymphocytes and some ASD etiological factors and markers associated with mitochondrial damage. **(A–C)** The interrelation between T-helper cells and some probable ASD etiological factors (Cytomegalovirus infection, VDR(Taq) SNP polymorphism, Lead content in packed RBC). VDR(Taq) SNP and the Lead content in packed RBC highly correlated with helper T-cells (*r* = 0.8757; *p* = 0.0020 and *r* = 0.9198; *p* < 0.0001, respectively). **(D–F)** The interrelation between T-helper cells and some analytes associated with mitochondria damage (HMG, QUIN, ASAT). A strong correlation was found between HMG, QUIN, ASAT and the content of T-helper lymphocytes (*r* = 0.8338; *p* = 0.0007, *r* = 0.8748; *p* = 0.0002, *r* = 0.7928; *p* = 0.0108). Pearson correlation coefficients were computed to determine inter-metabolite correlations. *P* < 0.05 were used to indicate statistical significance.

Three factors (infection, SNP, heavy metals) having etiological roles in the development of the autistic pathology were compared to the number of helper T-cells (CD3+/CD4+). As it was shown above, all patients were characterized with anti-CMV IgG appearance with high affinity. Nevertheless, we did not detect a statistically significant correlation between anti-CMV IgG values and CD3+/CD4+ cells numbers (*r* = −0.5191; *p* = 0.1521; Figure 9A). On the other hand, both VDR Taq SNP and the lead content in packed RBC highly correlated with helper T-cell numbers (*r* = 0.8757; *p* = 0.0020 and *r* = 0.9198; *p* < 0.0001, respectively; Figures 9B,C). Several metabolites associated with mitochondrial damage: HMG, QUIN, and ASAT were increased in ASD patients and strongly correlated with the changes in the content of T-helper lymphocytes (*r* = 0.8338; *p* = 0.0007, *r* = 0.8748; *p* = 0.0002, *r* = 0.7928; *p* = 0.0108; Figures 9D–F).

**Figure 10:**
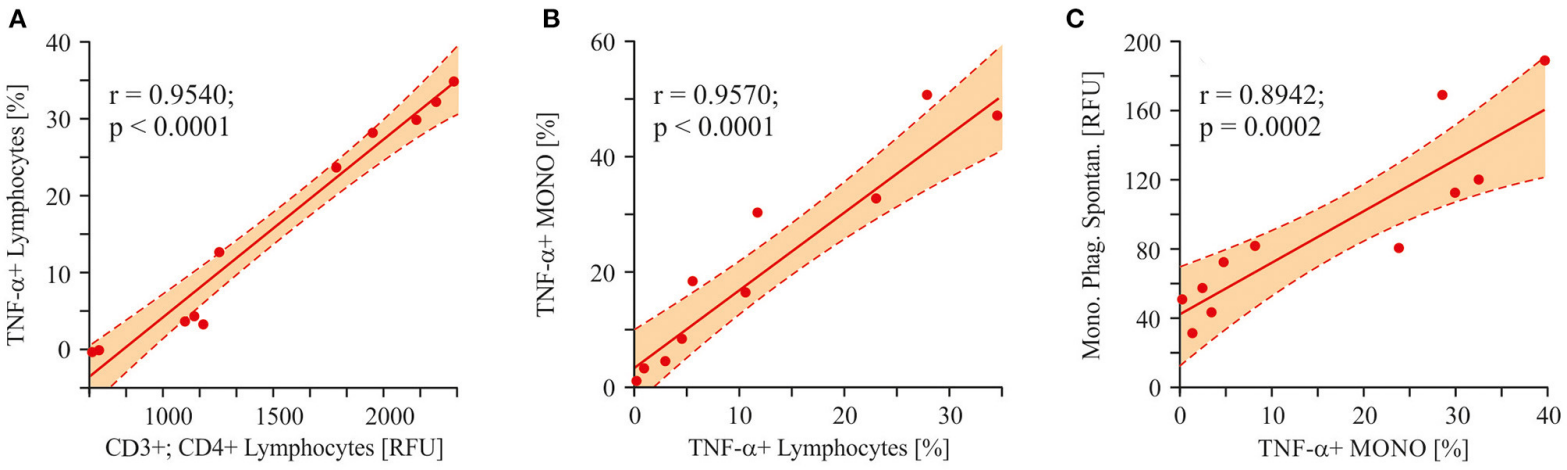
Linear regression analysis (Pearson product-moment correlation). The pathway of the effector cell (monocytes respiratory burst) activation. **(A)** The interrelation between T-helper cells and lymphocytes derived TNF-α (*r* = 0.9540; *p* < 0.0001). **(B)** The interrelation between lymphocytes derived TNF-α and monocytes derived TNF- α (*r* = 0.9570; *p* < 0.0001). **(C)** The interrelation between monocytes derived TNF-α and the spontaneous respiratory burst of the same cells (*r* = 0.8942; *p* = 0.0002). Pearson correlation coefficients were computed to determine inter-metabolite correlations. *P* < 0.05 were used to indicate statistical significance.

To trace the pathway of effector immune cell activation (monocyte phagocytosis) in autistic children, we analyzed the correlation between a pro-inflammatory cytokine (TNF-α) in different cell populations and the final effector property of monocytes—respiratory burst. Consequently, a strong correlation was shown between the levels of TNF-α in lymphocytes and CD3+/CD4+ T-cells (*r* = 0.9540; *p* < 0.0001; Figure 10A). Furthermore, lymphocyte and monocyte-derived TNF-α levels were also strongly correlated to each other (*r* = 0.9570; *p* < 0.0001; Figure 10B). And finally, a significant correlation was shown between monocyte TNF-α and the respiratory burst of the same cells (*r* = 0.8942; *p* = 0.0002; Figure 10C).

**Figure 11:**
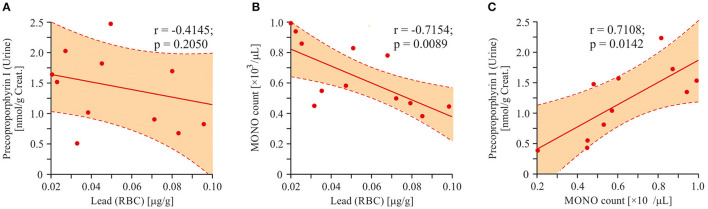
Linear regression analysis (Pearson product-moment correlation). Association between lead poisoning and precoproporphyrin appearance in urine of ASD patients. **(A)** The interrelation between precoproporphyrin and lead content (*r* = −0.4145; *p* < 0.2050). **(B)** The interrelation between monocytes count in blood and lead content (*r* = −0.7154; *p* = 0.0089). **(C)** The interrelation between precoproporphyrin and monocytes count in blood (*r* = 0.7108; *p* = 0.0142). Pearson correlation coefficients were computed to determine inter-metabolite correlations. *P* < 0.05 were used to indicate statistical significance.

We did not detect any statistically significant correlations between enhanced urine porphyrins level and lead content in RBC. In order to find a possible relationship between the urinary porphyrin levels and the increased concentration of lead, we attempted to find the porphyrins with the largest data scatter. Precoproporphyrin I was the best suited for this requirement (*SD* = 54.6%). Direct correlation between the values of lead and precoproporphyrin I was not found (*r* = −0.4145; *p* = 0.2050; Figure 11A). An attempt was made to find a factor mediating the dependence of the precoproporphyrin I level on the lead content. Inverse correlation between the lead content and monocyte count is shown in Figure 11B (*r* = −0.7154; *p* = 0.0089). However, a positive correlation was shown between monocyte count and precoproporphyrin I content (*r* = 0.7108; *p* = 0.0142; Figure 11C).

## Discussion

Our data describe autism pathology development by combining data on environmental toxicants, genetic predisposition, bioenergetics, and finally immune system over activation. Environmental metal and metalloid intoxications (lead, mercury, aluminum, and arsenic) are known as potential etiological factors leading to ASD (30). Indeed, we only observed enhanced levels of lead in packed RBCs of autistic children. Enhanced urine porphyrin content in autistic patients is typical for this pathology and is related to heavy metal intoxication (31–33). Here also, we showed disordered porphyrin metabolism, manifested predominantly by elevated concentrations of urinary coproporphyrins, total porphyrins, as well as uroporphyrines. However, we did not find any significant correlations between increased level of urinary porphyrins and lead concentration in RBC. At the same time abnormalities in porphyrin metabolism support the etiological role of heavy metal intoxication in ASD development in the matter of increased levels of lead and unchanged mercury and arsenic.

A simple complete blood count revealed the intoxication (diminished RBC parameters and enhanced RDW) and inflammation state (increase of the numbers of platelets, monocytes, neutrophils) in ASD children. The platelet count increase in ASD patients was shown earlier and this is typical for monogenetic and complex neurological diseases as several parallels exist between platelets and the brain cells (34–36).

Noteworthy are the diminished levels of vitamins B12 and D on the one hand, and enhanced ASAT on the other, in the blood serum of autistic children. A physiological level of ALAT with a simultaneously enhanced ASAT level indicates the extrahepatic origin of both enzymes. The ASAT is expressed in two forms: cytosolic (in the red blood cells and heart) and mitochondrial (liver, brain, etc.) (37). The cytosolic origin of the enhanced ASAT observed in our study can be excluded due to the physiological level of MCHC test shown in CBC data. This makes the mitochondrial origin of the increased ASAT in ASD patients more probable. As it was stated above, due to the low level of ALAT, its liver origin seems unlikely. On the other hand, the ASAT enzyme is assigned to important functions in astrocytes and neurons (38). In addition, as it was shown by Guidetti and coauthors (39), catalyzing the formation of the neuroinhibitory metabolite kynurenic acid, mitochondrial ASAT may be involved in a range of physiological and pathological processes associated with glutamatergic and nicotinergic signaling. Some studies demonstrate possible increase in permeability of blood-brain barrier (BBB) in ASD (40). Therefore, it can be speculated that neuronal cell damage and increased BBB permeability in ASD patients underlies the elevation of ASAT levels in blood serum.

Lead intoxication might be an etiological factor for neuronal cell damage and consequent ASD development. Negative association between blood lead levels and vitamin D/B12 concentrations has been reported (41–43). Probably, diminished vitamin D, among its classical Ca/P regulating role, would have much a more negative effect on immune system function in autistic children.

The most important form of vitamin D, cholecalciferol (vitamin D3), is known to stimulate differentiation of immune cells (44, 45). This concept was supported by observations that showed different expressions of the vitamin D receptor (VDR) and α-1-hydroxylase at the different stages of differentiation of macrophages. Some studies show that human macrophages are able to synthesize 1,25(OH)_2_D3 upon exposure to IFNγ (43–46). VDR receptor gene polymorphisms were identified in various diseases as shown in reviews of Valdivielso and Fernandez (47) and Uitterlinden et al. (48). Polymorphisms in Bsm-I, Taq-I, Apa-I, and Fok-I were associated with renal diseases, cancer, neurolithiasis, and diabetes. In addition, some authors showed correlation between VDR gene polymorphisms and susceptibility to asthma and atopic dermatitis (49, 50). Abnormalities in the vitamin D receptor, and low levels of vitamin D were both linked to Parkinson's disease and autism (50–52). It is known that lead exposure may be associated with increased risk of ASD (41, 42). Polymorphisms in the genes coding for VDR may affect susceptibility to lead exposure (53). Our results convincingly demonstrate the enhanced ratio of *T* allele at position Taq-I (rs731236) (*OR* = 3.71) and of the VDR gene in autistic children. The ratio of *T* allele at position Fok-I (rs2228570) was not significantly different in ASD patients. Association between VDR Taq polymorphism and susceptibility to ASD was shown earlier (52, 54).

As it was stated above, vitamin D3 plays a crucial role in the development and function of the brain (55), and vitamin D can therefore be implicated in neuropsychiatric disorders, such as autism spectrum disorder (56). By interaction with the specific VDR, the developmental and functional consequences of vitamin D in the nervous system can be modulated. It was shown that patients bearing mutations in their VDR receptor gene might have a different activation threshold than the wild form of the receptor (57, 58).

In addition, ASD patients have been described with enhanced ratios (*OR* = 4.89) of the *T* allele of monoamine oxidase A (MAO-A R297R-*GT*). It is known that children with the low-activity MAO-A allele have both lower intelligence quotients and more severe autistic behavior than children with the high-activity allele (59, 60). This is due to the diminished activity of MAO-A leading to decreased serotonin degradation and its accumulation in the brain. This has long been implicated in the psychopathology of autism (60, 61). Such low MAO-A activity leading to serotonin accumulation is also expected to cause decreases in 5-hydroxyindolacetate (5-HIAA) levels which, nevertheless, were not observed in our study. It was also shown that the activation of the kynurenine pathway (KP) of tryptophan degradation in neuroinflammation results in reduced serotonin synthesis from tryptophan and production of KP metabolites (62). Indeed, we have demonstrated the enhanced contents of QUIN and picolinate in ASD patients, which might indicate the overactivation of kynurenine pathway of tryptophan catabolism. The pathological levels of QUIN are associated with numerous neurological diseases: Alzheimer's disease, anxiety, depression, epilepsy, ASD, etc. (63, 64). Moreover, generation of QUIN is thought to be the major link between the KP and inflammatory response (65). The first enzyme of the KP, indoleamine 2,3-dioxygenase (IDO-1), is induced by various proinflammatory cytokines (63). So, in immune-activated states, IDO-1 may catabolize a large proportion of tryptophan leading to shortage for the serotonin–melatonin pathway. Also, the increased levels of QUIN in the brain could alter the excitation/inhibition ratio of the N-methyl-D-aspartate receptor, ultimately leading to excitotoxicity. Hence, QUIN may act as an endogenous excitotoxin that contributes to the pathogenesis of ASD, especially during neuroinflammation (60).

Indeed, the presence of a proinflammatory environment in ASD pathology is described in this paper. We have observed upregulation (in peripheral blood leukocytes) of TNF-α, which is known as one of the major proinflammatory cytokines involved in the pathogenesis of different diseases (64). At the same time, the level of the anti-inflammatory cytokine, IL-10, was also increased, indicating the existence of a probable negative feedback loop (66, 67). In addition, immune cell phenotype analysis showed elevation in T-cell (CD3, CD4, and CD8) count and monocyte phagocytosis increase. In line with the latter, the analysis of respiratory burst (spontaneous and fMLP-induced) of peripheral blood monocytes in autistic patients revealed enhancement of spontaneous phagocytosis and unaltered fMLP-induced phagocytosis. It has been reported by different authors and also by us that mitochondrial dysfunction observed in autistic children was accompanied by a lower oxidative burst in the phorbol-12-myristate-13-acetate (PMA)-stimulated granulocytes (13, 68), that is also confirmed by the current study. In monocytes obtained from peripheral blood of autistic patients we did not observe characteristic right shift of Rhodamin 123 fluorescence upon induction by fMLP. This suggests that the persistent inflammation shown in ASD patients may lead to the depletion of respiratory burst capability in neutrophils and accumulation of damage and pathological changes resulting in disability and disease (69).

In this study we showed activation of adaptive immunity, which manifested by an increase in T cell count, and even slight down regulation of innate immunity, which, in turn, was expressed by a decrease in level of NK cells. Interestingly, the elevation of T-cells was accompanied by an increase of TNF-α production in monocytes, macrophages (CD14+), and T-lymphocytes (CD3+). A systematic review by Mitchell R. and Goldstein B. documented preliminary evidence of the association of pro-inflammatory markers in almost 4,000 children with neuropsychiatric and neurodevelopmental disorders, including ASD (70). Like others we also suggest that a feasible mechanism for the role of inflammation in ASD is the violation of functional integrity of the CNS by cytokines, thereby contributing to the neuroinflammation (71). There are three main pathways for peripheral cytokines and their mediated signals to access the brain—humoral, neural, and cellular (72). In the humoral pathway, cytokines cross the BBB through leaky regions (choroid plexus and circumventricular organs); with the neural pathway, activated monocytes and macrophages stimulate primary afferent nerve fibers of the vagus nerve. For the cellular pathway, it is suggested that cytokines, principally TNF-α, could stimulate microglia to recruit monocytes into the brain (possibly via activation of the production of monocyte chemoattractant protein-1) (72, 73). Thus, based on the literature data and our findings, it can be suggested that TNF-α produced by activated monocytes and macrophages initiates and/or mediates inflammation in the brain of ASD patients. We are prone to believe the humoral mechanism of cytokine access to the brain in ASD. In this regard the possible increase of BBB permeability in ASD (40) also supports our thoughts. At the same time, we do not exclude that inflammation develops directly in the brain through glial cells, independently of peripheral events or in addition to them.

Oxidative stress resulting in overproduction of ROS is a well-known factor in the development of inflammatory response (74). The role of ROS in pathogenesis of ASD was discussed in our previous study (13). Here, we have focused on the factors leading to the abnormal ROS production and chronic inflammation in ASD, such as energy production machinery, which probably leads to mitochondrial dysfunction and chronic inflammation in ASD patients.

Increased QUIN and picolinate observed in our study apparently indicate decreased activity of quinolinic acid phosphoribosyltransferase (QPRT). QPRT is an important enzyme in the kynurenine pathway (KP) which regulates the intracellular NAD^+^ synthesis in human astrocytes and neurons. The NAD^+^ levels are mainly dependent on the KP metabolism and Indoleamine-pyrrole 2,3-dioxygenase (IDO) and QPRT regulation (75, 76). Thus, anticipated inhibition of QRPT should lead to the observed QUIN increase and NAD^+^ decrease. Altered QUIN levels could also result in altered NAD^+^ biosynthesis, which in turn may affect the poly (ADP-ribose) polymerase (PARP) activity leading to neuronal cell death (77). Moreover, it has been suggested that QPRT protein acts as an inhibitor of spontaneous cell death by suppressing overproduction of active-caspase-3. The inhibition of caspase-3 activity/synthesis or posttranslational modification might have a pro-apoptotic effect and lead to neuronal cell death (78). It has also been demonstrated that the autonomous cell generation of NAD^+^ via the KP regulates macrophage immune function in aging and inflammation. Isotope tracer studies revealed that macrophage NAD^+^, to a large extent, depends on the KP metabolism of tryptophan. Genetic or pharmacological blockade of the *de novo* NAD^+^ synthesis results in NAD^+^ depletion, suppressed mitochondrial NAD^+^-dependent signaling and respiration, and impaired phagocytosis and resolution of inflammation as discussed above (79).

It is known that NAD^+^ pools can modulate the activity of compartment-specific metabolic pathways, such as glycolysis in the cytoplasm and the citric acid cycle cycle/oxidative phosphorylation in mitochondria (80). It is also well-known that the citric acid cycle is the main electron donor (in the form of NADH) for mitochondrial respiratory chain. Any hindrance in the electron flow might bring about ROS overproduction, mitochondrial dysfunction, and oxidative stress. All these aberrations have been observed in autistic pathology. In order to evaluate the function of the citric acid cycle, we have measured the main analytes of this pathway and demonstrated the increase of Cis-Aconitate, Isocitrate, and α-ketoglutarate. This might indicate the inhibition of the α-ketoglutarate converting reaction catalyzed by oxoglutarate dehydrogenase (OGD). OGD catalyzes the conversion of α-ketoglutarate to succinyl CoA, with reduction of NAD+ to NADH. OGD is a key regulator in the citric acid cycle and is inhibited by succinyl-CoA and NADH. OGD is considered to be a redox sensor in the mitochondria. Increased NADH/NAD+ ratio is associated with enhanced ROS production and inhibited OGD activity (81, 82). Thus, diminished NAD^+^ *de novo* synthesis could lead to lower NAD^+^/NADH ratio, mitochondrial dysfunction, and oxidative stress (83).

We have also observed HMG increase in autistic patients. HMG is a metabolite related to the energy production pathway and cholesterol synthesis. A high HMG level might indicate insufficient Coenzyme Q10 (CoQ10) production resulting in the leakage of ROS (84). CoQ10 is considered an effective endogenously synthesized lipid soluble antioxidant, which inhibits oxidative stress and overactive inflammatory response by regenerating vitamin E or by quenching superoxides or another ROS. CoQ10 is a key component of the oxidative phosphorylation process of the mitochondrial respiratory chain. Apart from its anti-oxidative function, CoQ10 also appears to modulate immune functions (85–87).

Inflammatory markers, such as IL-1β and TNF-α, were shown to increase in the brains of many ASD patients (88). Apparently, this could be compared with the enhanced TNF-α in monocytes and lymphocytes of autistic patients and simultaneously enhanced IL-10 produced by monocytes and CD 14+ cells only, as shown in the current study.

It can be suggested that activated inflammatory pathways, oxidative stress, mitochondrial dysfunction, and brain metabolic disorders are involved in the ASD pathophysiology. We have attempted to address the question of if there is any correlation between these pathways altered in ASD and which of them is more important in the development of this pathology. Correlation analyses suggest that the main factor(s) in the development of ASD is T-helper (CD4+ CD3+) lymphocytes. The increase in the levels of these cells strongly correlates both with etiological factors (VDR-Taq polymorphism and Lead poisoning) and with pathogenic factors (HMG, QUIN, and ASAT). Based on this analysis, we propose the following scenario for the development of a chronic inflammatory response in autism. Lead intoxication, the effect of which is augmented by a mutation of the VDR-Taq, leads to mitochondrial dysfunction and, therefore, ROS overproduction. The latter were shown to activate the T-cell dependent immune response (89). We have also observed HMG increase in autistic patients. HMG is a metabolite related to the energy production pathway and cholesterol synthesis. A high HMG level might indicate insufficient Coenzyme Q10 (CoQ10) production resulting in the leakage of ROS (90). CoQ10 is considered an effective endogenously synthesized lipid soluble antioxidant, which inhibits oxidative stress and overactive inflammatory response by regenerating vitamin E or by quenching superoxides or another ROS. CoQ10 is a key component of the oxidative phosphorylation process of the mitochondrial respiratory chain. Apart from its anti-oxidative function, CoQ10 also appears to modulate immune functions (91–93).

**Figure 12:**
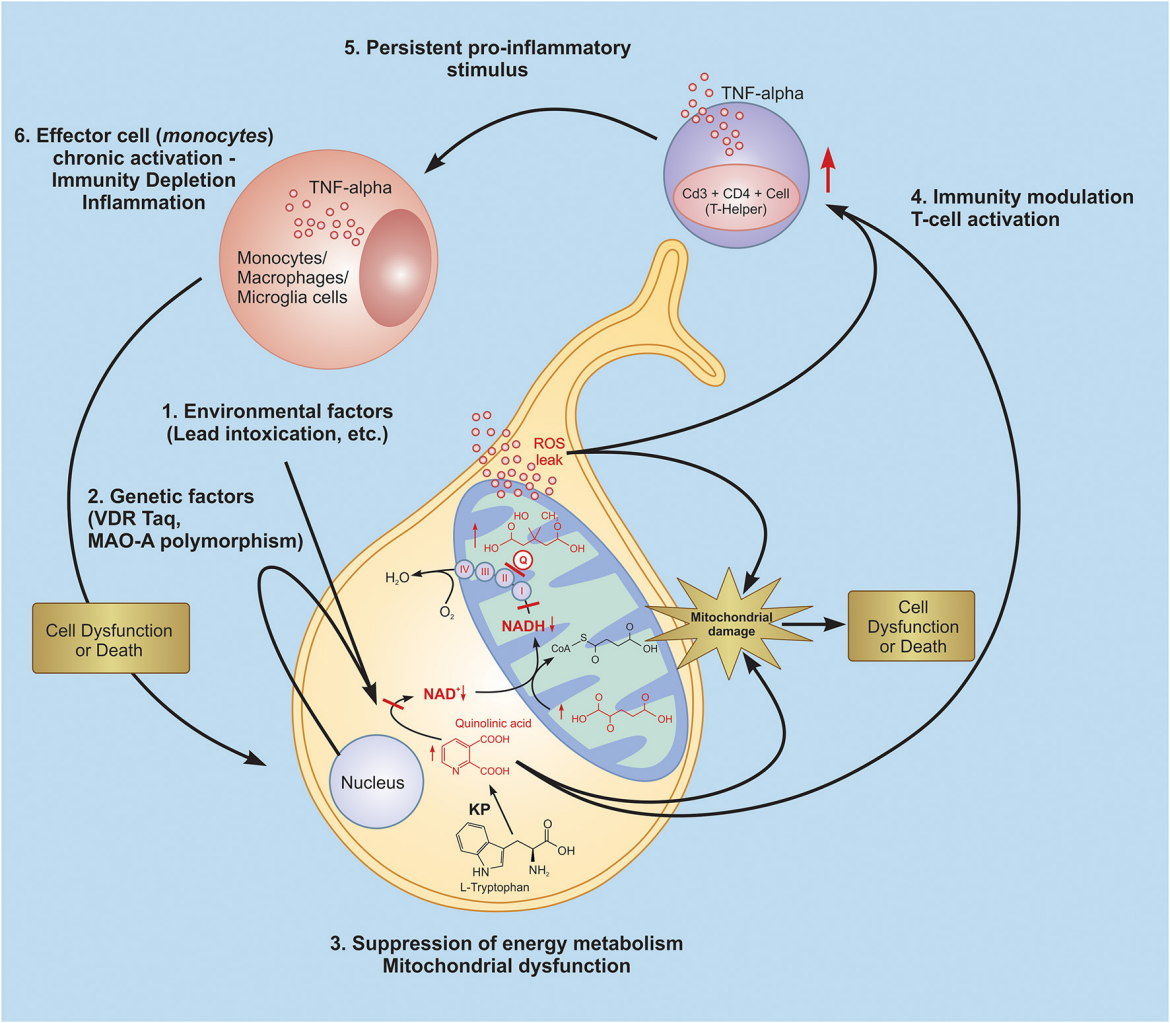
Probable glance at inflammatory scenario development in autistic pathology. Lead intoxication, the effect of which is intensified by a mutation of the VDR-Taq and MAO-A leads to quinolinic acid increase, resulting in energy metabolism depletion and mitochondrial dysfunction, which is expressed in ROS overproduction. The latter are also known to be signal-trigger molecules which activate the T-cell dependent immune response. Activated T-helpers produce pro-inflammatory cytokines (TNF-α), which leads to the activation of effector cells - macrophages and the development of a chronic inflammatory response and induce persistent inflammatory signals. KP, kynurenine pathway; Q, coenzyme Q10.

Inflammatory markers, such as IL-1β and TNF-α, were shown to increase in the brains ofmany ASD patients (94). Apparently, this could be compared with the enhanced TNF-a in monocytes and lymphocytes of autistic patients and simultaneously enhanced IL-10 produced by monocytes and CD 14+ cells only, as shown in the current study. It can be suggested that activated inflammatory pathways, oxidative stress, mitochondrial dysfunction, and brain metabolic disorders are involved in the ASD pathophysiology. We have attempted to address the question of if there is any correlation between these pathways altered in ASD and which of them is more important in the development of this pathology. Correlation analyses suggest that the main factor(s) in the development of ASD is T-helper (CD4+ CD3+) lymphocytes. The increase in the levels of these cells strongly correlates both with etiological factors (VDR-Taq polymorphism and Lead poisoning) and with pathogenic factors (HMG, QUIN, and ASAT). Based on this analysis, we propose the following scenario for the development of a chronic inflammatory response in autism. Lead intoxication, the effect of which is augmented by a mutation of the VDR-Taq, leads to mitochondrial dysfunction and, therefore, ROS overproduction. The latter were shown to activate the T-cell dependent immune response (95). Activated T-helpers produce pro-inflammatory cytokines (TNF-α), which lead to the activation of effector cells-macrophages and the development of a chronic inflammatory response (Figure 12).

## Limitations

Several limitations of the study should be noted. All metabolites' measurements were done in blood and urine; nevertheless, it would be better to measure some of them in the brain and CSF also. However, due to obvious barriers to getting such material we limited ourselves to the data obtained from the specified samples. Next, we acknowledge that our sample size was somewhat small and may have limited the power to detect subtle group differences and associations. However, blood sample collection in young children under the age of 6 years and, in particular with neurodevelopmental disorders, is an intractable issue, especially taking into account the small population in Armenia. In spite of the small sample size in the present study, observed data might be generalizable due to target statistical approach and the huge range of measurements, both of which enhance the statistical power of the study. On the other hand, based on our current findings better-controlled studies with larger sample sizes have to be designed and conducted.

## Conclusion

In summary, our study suggests a possible scenario of the development of autistic pathology (see Figure 12). Lead intoxication, as a potential etiological factor, and VDR Taq and MAO-A polymorphisms, as potential risk factors, trigger ASD development. As proven, the aforementioned factors invoke disturbances in the functioning of such key cellular systems as the kynurenine pathway, the citric acid cycle, and mitochondrial respiratory chain, leading to mitochondrial dysfunction and ROS overproduction. Our findings argue the latter brings about the T-cell (CD4+CD3+) dependent immune system activation and persistent chronic inflammatory response in ASD patients.

## Data Availability Statement

The raw data supporting the conclusions of this article will be made available by the authors, without undue reservation. The deposited data can be found in the following repository: https://www.ncbi.nlm.nih.gov/SNP/snp_viewTable.cgi?handle=YSMU.

## Ethics Statement

The studies involving human participants were reviewed and approved by Yerevan State Medical University After Mkhitar Heratsi, Yerevan, Armenia. Written informed consent to participate in this study was provided by the participants' legal guardian/next of kin.

## Author Contributions

AH: investigation, methodology, analysis, and writing—original draft. HH: investigation, methodology, analysis, writing—original draft, and writing—review and editing. KY: conceptualization, methodology, investigation, analysis, supervision, writing—original draft, writing—review and editing, and funding acquisition. All authors contributed to the article and approved the submitted version.

## Funding

This work was supported by the YSMU, the State Committee of Science RA (N 10-14/I-1 and 20TTCG-3A012), and EU funded H2020 COBRAIN project (857600).

## Conflict of Interest

The authors declare that the research was conducted in the absence of any commercial or financial relationships that could be construed as a potential conflict of interest.

## Publisher's Note

All claims expressed in this article are solely those of the authors and do not necessarily represent those of their affiliated organizations, or those of the publisher, the editors and the reviewers. Any product that may be evaluated in this article, or claim that may be made by its manufacturer, is not guaranteed or endorsed by the publisher.
